# Working in the Norwegian School Health Services: A Quantitative Cross-Sectional Study of School Nurses’ Work Situation

**DOI:** 10.1177/10598405251327913

**Published:** 2025-03-24

**Authors:** Gunhild Hustad, Marit Müller De Bortoli, Elisabeth Holm Hansen

**Affiliations:** 1 Department of Nursing and Health Sciences, 6660 University of South-Eastern Norway, Porsgrunn, Norway; 2 Department of Child and Adolescent Health Promotion Services, 25563 Norwegian Institute of Public Health, Levanger, Norway; 3 155312 VID Specialized University, Bergen, Norway

**Keywords:** school health services, school nurse, cross-sectional study, national professional guidelines, work environment, collaboration, children, adolescents, school nursing

## Abstract

As school health services continue to evolve, it becomes increasingly important to highlight the work situation of school nurses. This study explored the role of school nurses within Norwegian school health services using a web-based questionnaire that was completed by 270 school nurses in a cross-sectional study. The survey focused on the National Professional Guidelines, cooperation with schools, the physical work environment, and job satisfaction. The findings revealed that more years of experience led to better inclusion by school staff, and having a colleague reduced feelings of isolation. Many school nurses were satisfied with their jobs and valued their work. However, less than half found the National Professional Guidelines to be clear regarding expected activities. These insights highlight areas for potential improvement in school health services.

## Background

School health services play an important role in the promotion of good health among school-aged children worldwide (WHO, 2021). The Norwegian school health services are available to children and adolescents where they spend their daily time and are free of charge. This accessibility removes barriers such as transportation logistics and appointment scheduling, thereby making it a service that reduces social disparities (Norwegian Directorate of Health, 2017b; WHO, 2021). The primary objective of the school health services is to work preventively and promote health (Norwegian Directorate of Health, 2017b).

Over the past decade, Norway has significantly invested in the school health services by hiring more school nurses with governmental support (NOU, 2023, p. 4), which reflects the political emphasis on preventive healthcare for children and adolescents (Norwegian Directorate of Health, 2021). Compared to other countries in Europe, Norway spends a considerable amount of money on school health services (van der Pol et al., 2020).

In the context of school health services, the term “school nurse” is used internationally. In Norway, the primary professionals in school health services are nurses with either a post-bachelor's degree or master's degree (Norwegian Ministry of Education and Research, 2021). The Norwegian term for nurses with this extended education is “health nurses.” This term emphasizes the primary focus of their work, which is centered on health promotion rather than disease management or caregiving (Norwegian Directorate of Health, 2017b). The most equivalent term in English is public health nurse (PHN). Norwegian PHNs work in health centers for children (0–5 years), school health services, and youth health centers for adolescents up to 20 years of age, and often work across all these areas (Norwegian Directorate of Health, 2017b).

According to Norwegian law, school health services shall be staffed by educated PHNs. However, there is a generally shortage of educated PHNs (Meld.St.23 (2022–2023)), and educational institutions also face capacity issues, with many applicants for limited places (Helleve et al., 2020; Lassemo & Melby, 2020). Due to these staffing challenges, many municipalities temporarily hire nurses without PHN education, often in part-time or temporary positions (Helleve et al., 2020; Lassemo & Melby, 2020).

In Norway, municipalities are legally required to provide health services at schools, which are governed by the Health and Care Services Act (Norwegian Ministry of Health and Care Services, 2018) and regulated by the National Professional Guidelines (hereafter Guidelines) (Norwegian Directorate of Health, 2017b). Although municipalities have autonomy in organizing the services, they must adhere to the Act and the Guidelines (Norwegian Directorate of Health, 2017b). The Guidelines include a chapter on school health services, which serves as a job description for school nurses, aiming to standardize practices and ensure fair prioritization. In the terminology used in the Guidelines, “shall,” “should,” and “may” indicate the level of obligation, from statutory requirement to strong and mild recommendations (Norwegian Directorate of Health, 2017b).

The Norwegian school systems are regulated by the Education Act. A new Education Act was published in 2023 (Norwegian Ministry of Education and Research, 2023), which only mentions school health services once, stating that those working in schools, including school health services staff, are prohibited from covering their faces while at work; beyond this, the school health services are not referred to. Despite being governed by separate legislative Acts, schools and school health services share the primary aim of promoting health, well-being, and a good learning environment for children and adolescents (Norwegian Directorate of Health, 2017b; Norwegian Ministry of Education and Research, 2023).

School nurses must share their workplace and cooperate with school staff. The Guidelines require school nurses to attend meetings and participate in school development days. If such cooperation is absent, school nurses must take the initiative to establish a formal cooperation (Norwegian Directorate of Health, 2017b). Strong individual relationships and physical proximity enhance cooperation (Helleve et al., 2020). Being centrally located within the school increases opportunities for informal dialogue and ad-hoc meetings, thereby facilitating collaboration (Clancy et al., 2013; Waldum-Grevbo, 2018).

In addition to good cooperation a good physical work environment is important for individuals, thereby promoting health and enhancing job satisfaction (Almenningen et al., 2024). The previous version of the Education Act (Norwegian Ministry of Health and Care Services, 1984) required municipalities to provide school nurses with adequate office space and facilities. However, this provision is not included in the current Act or Guidelines (Norwegian Directorate of Health, 2017b; Norwegian Ministry of Health and Care Services, 2018).

In the legal framework for schools, a provision exists for the school to cooperate with others, specifically in the context of individual pupils who require comprehensive and coordinated health services. However, schools are not required by the Education Act to cooperate with the school health services (Norwegian Ministry of Education and Research, 2023). This discrepancy in wording and obligations between the Education Act and the Guidelines is a concern for school nurses (Waldum-Grevbo, 2018).

Research in Norway and internationally has examined the challenges faced by school nurses and their job satisfaction. Studies from the USA and Norway have underscored that case complexity, technological advancements, and staffing shortages increase work demands (Lassemo & Melby, 2020; Morse et al., 2022; Nygård et al., 2024). High workloads expose school nurses to a greater risk of burnout (Jameson & Bowen, 2020). Additionally, having a manager without a healthcare background can cause stress due to a lack of understanding of school health services (Foley et al., 2004).

Among all health-educated groups, school nurses in Norway have the lowest turnover rate in terms of employment (NOU, 2023), which suggests the existence of specific factors that influence their decisions to remain in their jobs. A qualitative study identified some of these factors, such as the ability to maintain authenticity in their work and making independent decisions (Mæland et al., 2021). These findings have been supported by other studies, showing that autonomy is an important component in the daily work of school nurses (Federici et al., 2021; Foley et al., 2004).

School health services are evolving, and in recent years, several suggestions for changing their purpose and content have been made. For instance, the Norwegian government has proposed that school health services should also take on responsibilities such as the diagnosis, treatment, and follow-up of pupils (Norwegian Ministry of Health and Care Services, 2017, 2020). There has also been a proposal to investigate a merging of the educational-psychological services (EPS) with school health services (Meld. St. 6 (2019–2020)). In this context, it is crucial for school nurses to highlight their work, articulate their needs, and propose the best direction for their services (Morse et al., 2022). Understanding the current work situation of school nurses is essential to facilitate this communication.

The aims of this study were to analyze the work situation of school nurses with an emphasis on the Guidelines, cooperation with the school, the physical work environment, and job satisfaction.

## Methods

### Design and Setting

The research objectives were addressed using an electronic questionnaire. The study was set in the Norwegian school health services, which encompass obligatory elementary schools (1st–7th grade) and middle schools (8th–10th grade), and optional high schools (which in Norway lasts for 3–4 years). In Norway, children start school in the year that they turn 6 years old.

### Recruitment and Participants

Invitations to participate in the survey were distributed to 120 municipalities across three counties in eastern Norway. The information was sent via email to all leaders of school health services in these municipalities, who were asked to forward the questionnaire to their staff. To determine the number of school nurses who received the invitation, leaders were requested to report back the number of employees they had forwarded the email. However, due to the lack of response to this request, the exact number of recipients and the response rate are unknown.

The inclusion criteria for this study were school nurses who worked full or part-time in school health services in the selected area, and were employed in elementary school, middle school, and/or high school. Participation in the study was voluntary. Participants provided their written informed consent and were given the option to withdraw from the study up until the data analysis stage.

The study was granted approval by the Norwegian Agency for Shared Services in Education and Research on May 10, 2023, reference number 263032.

### Data Collection

As no pre-existing questionnaire was found to answer the research questions, a new one was developed. The questionnaire was created in collaboration with five expert groups, with two to six school nurses working in school health services participating in each group, totaling 17 school nurses. Ten individual school nurses from four different municipalities pilot-tested the questionnaire. None of these individuals participated in the final survey. Based on the feedback from the pilot test, adjustments were made to the questionnaire, including changes to the phrasing of some questions that could be misunderstood and revision of the response options. The final questionnaire was created using the digital tool; Nettskjema (University of Oslo, 2024). Data collection took place from June to September 2023.

### Measures

Given that many school nurses work across multiple schools and at various educational levels, respondents were asked to answer questions specifically about the last school that they had worked at. An online questionnaire consisting of 35 questions and 10 fields for comments was used. Data from the comments fields are not reported in this article. The background variables included in this article are presented in Table 1. Most questions used a Likert scale, with the following possible answers: “not at all,” “to a small extent,” “to some extent,” “to a large extent,” and “to a very large extent.”

**Table 1.** Participant Characteristics.

	n	%
**Age n = 268**		
20–30 years	14	5.2
31–40 years	66	24.4
41–50 years	115	42.6
51–60 years	63	23.3
61 + years	10	3.7
**Years of experience in school health services n = 270**		
0–2 years	54	20.0
3–6 years	90	33.3
7 + years	126	46.7
**Highest education level n = 270**		
Bachelor's degree (3 years at university or college)	27	10.0
Postgraduate certificate in PHN	222	82.2
Master's degree	21	7.8
**Number of schools served n = 270**		
1 school	171	63.3
2 schools	74	27.4
3–6 schools	25	9.3
**Working in education levels n = 270**		
Elementary school	144	53.3
Middle school	45	16.7
High school	20	7.4
Multiple levels^a^	61	22.6
**Team structure**		
Working with other school nurses	101	37.4
Working as the only school nurse	168	62.2

^a^ “Multiple levels” refers to working across multiple school levels.

PHN: public health nurse.

**Figure 1.**
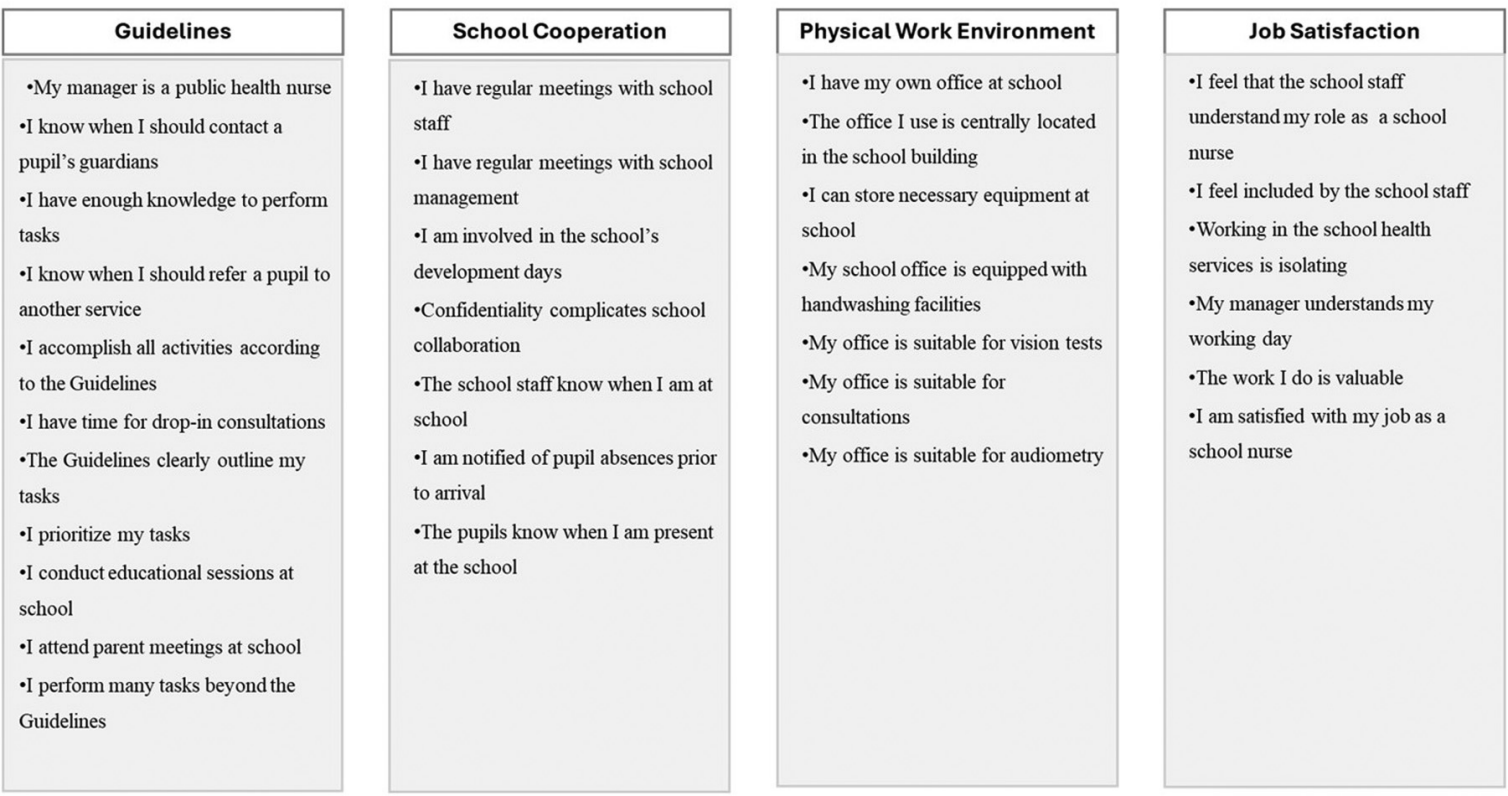
Overview of the contents within each theme addressed in the survey.

The primary theme of the questionnaire was the school nurses’ work situation in the school health services. The questions were categorized into four focus areas: guidelines, collaboration, physical work environment, and job satisfaction (Figure 1). Some questions could fall under two categories. For example, meetings with school staff could be considered both as an activity that school nurses should perform according to the Guidelines and as a question about cooperation with the school.

### Data Analysis

The data analysis in this study was carried out using IBM SPSS software (version 27.0) for Windows. The focus of the analysis was to understand the work situation of school nurses in providing health services in schools. The main themes and the contents within each one addressed in the survey were developed in collaboration with the expert groups. The survey focused on the work situation of school nurses, with an emphasis on the National Guidelines, cooperation with the school, the physical work environment, and job satisfaction. Descriptive statistics were used as a tool to examine these areas.

The association among three factors related to job satisfaction was evaluated using chi-square tests. For these analyses, both dependent and independent variables were dichotomized. The first two factors examined the correlation between experience and the sense of being understood and included by the school staff. The independent variable “length of experience” was dichotomized into “short” (0–4 years) and “long” (≥ 5 years). The dependent variables represented “low” (responses of “not at all” and “to a small extent”) and “high” (responses of “to some extent,” “to a large extent,” and “to a very large extent”).

Another correlation to be evaluated was between the feeling of isolation and having a colleague. The dependent variable was the level of perceived isolation, dichotomized into “low isolation” (including “not at all” and “to a small extent”) and “high isolation” (including “to some extent,” “to a large extent,” and “to a very large extent”). The independent variable in this analysis was team structure, indicating whether school nurses worked individually or with a colleague.

## Results

Table 1 presents the characteristics of the participants. The survey included responses from 270 school nurses across 71 municipalities, with a mean age of 45 years. Due to the minimal representation of men (six respondents), gender was excluded as an independent variable. Most respondents worked at elementary schools (n = 144), which is likely to be because these schools have more grades and pupils. Additionally, many participants reported working at more than one school level (n = 61).

### Survey Results

#### Guidelines

**Figure 2.**
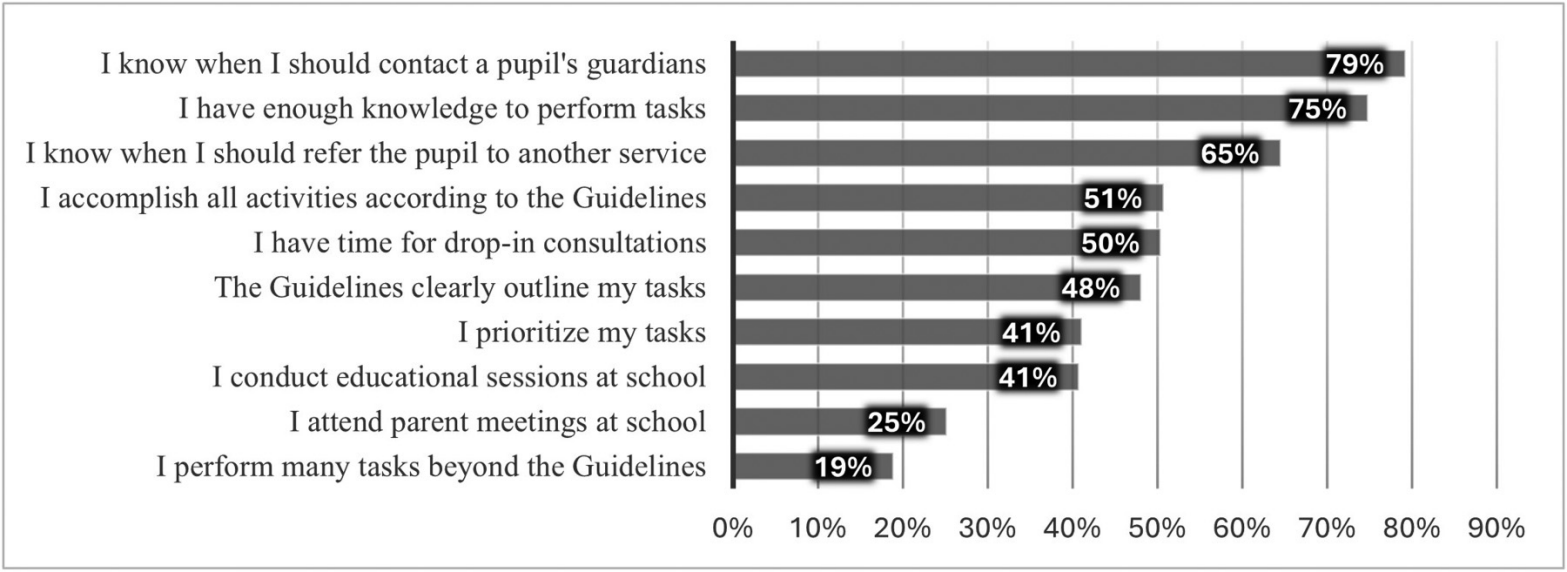
Percentage of respondents who answered “to a large extent” and “to a very large extent.”

The survey included questions regarding whether the school nurses follow the Guidelines. A specific point in the Guidelines is that the professional leader of the school health services should be an educated PHN (Norwegian Directorate of Health, 2017b). Only 5% responded negatively to this question, which indicates that nearly all municipalities have a leader who is an educated PHN. Figure 2 presents the percentage who responded “to a large extent” or “to a very large extent” to questions regarding the Guidelines.

In the survey, respondents were asked if the Guidelines clearly outline their tasks in school health services. More than 50% of the respondents indicated that the Guidelines regarding their activities within school health services were “not at all clear,” “clear to a small extent,” or “clear to some extent.” This indicates that a large proportion of respondents found the Guidelines to be unclear. There were no differences based on educational level; both school nurses with PHN education and those without perceived the clarity of the Guidelines similarly. The group that found the Guidelines most unclear was those working in high schools, with 70% of these indicating that they found the Guidelines unclear.

Most respondents felt that they had sufficient knowledge, with slightly less than a quarter feeling that they lacked adequate knowledge to some extent. This finding was consistent across elementary, middle and high school.

Participation of school nurses in parent meetings was found to be lowest at the high school and elementary school levels, with approximately 15% reporting high participation in these meetings. Conversely, 54% of middle school nurses reported a high level of participation in parent meetings.

One of the survey questions asked school nurses to identify the themes that they teach in school as outlined in the Guidelines (they could select multiple topics). The results were as follows, with the percentage indicating the proportion of respondents who reported teaching each theme: mental health (86.2%), puberty (82.8%), food and nutrition (55.6%), sexual health (63.8%), sleep hygiene (51.9%), physical activity (49.3%), and tobacco, alcohol, and drugs (24.6%).

#### Cooperation

Seven questions were included in the category aimed at evaluating cooperation between school nurses and the school, as experienced by the school nurses. Slightly more than half of the school nurses had meetings with school management (52.6%); however, a smaller proportion had meetings with school staff (37.4%). Additionally, not many participated significantly in the school's development days (16.3%). School nurses reported that the school staff knew when they were present at school (89.3%); however, the school nurses were rarely informed when a pupil was absent (10.4%). The results are presented in Table 2, with the five possible answers condensed into three categories.

**Table 2.** Cooperation with the School.

	Not at all / to a small extent		To some extent		To a large extent /to a very large extent	
	n	(%)	n	(%)	n	(%)
I have regular meetings with school staff	87	(32.2)	82	(30.4)	101	(37.4)
I have regular meetings with school management	50	(18.5)	78	(28.9)	142	(52.6)
I am involved in the school's development days	126	(46.7)	100	(37.0)	44	(16.3)
Confidentiality complicates school cooperation	86	(31.9)	157	(58.1)	26	(9.7)
The school staff know when I am at school	1	(0.4)	25	(9.3)	241	(89.3)
I am notified of pupils’ absences prior to arrival	183	(67.8)	58	(21.5)	28	(10.4)
The pupils know when I am present at the school	1	(0.4)	41	(15.2)	226	(83.7)

Some rows do not add up to 270 (100%) due to missing data.

#### Physical Work Environment

The work environment refers to the physical conditions and the arrangements that are made for school nurses to perform their activities within school health services. Nearly all (96%) school nurses reported having their own office at school, with 90% stating that they could store their equipment at school and 12% reporting not having a centrally located office within the school building. When asked if the office was suitable for conducting hearing and vision tests, just over half responded positively. Regarding consultations, 72% reported that their office was well-suited for this purpose. Furthermore, when asked if they had handwashing facilities in their office, 32% stated that they did not.

#### Job Satisfaction

The survey included six questions regarding work satisfaction in school health services. Among these, three questions had a high percentage of similar responses: “My manager understands my working day,” (77%), “The work I do is valuable” (84%), and “I am satisfied with my job” (87%), where the percentages refer to those who answered, “to a large extent” or “to a very large extent.” Due to this high level of agreement, there was not enough variation to perform meaningful statistical analyses on this dependent variable.

A chi-square test was conducted with the length of experience as the independent variable and feeling understood and included by the school staff as the dependent variable. The results indicated a correlation between the length of experience and feeling included, with school nurses who had more experience feeling more included by the school staff than those with less experience (Table 3).

**Table 3.** Association Between Experience and Role Understanding and Inclusion.

	Total N = 270		Experience		p-value^a^
Dependent variable	n	(%)	Short 0–4 years	Long ≥5 years	
I feel that school staff understand my role as a school nurse	268	(99.3)			
Low (reference)	121	(44.8)	52	69	0.075
High	147	(54.4)	47	100	
I feel included by the school staff	270	(100)			
Low (reference)	108	(40)	52	56	0.003^b^
High	162	(60)	49	113	

^a^ p-value exact Sig.(2-sided); ^b^significant at the 95% confidence level.

The question about whether the school nurses felt isolated in their roles revealed that 20% felt isolated to a large or very large extent, which increased to 70% when including those who reported feeling “some” isolation. A chi-square test, with team structure as the independent variable and perceived isolation as the dependent variable, indicated that working with a colleague significantly reduced feelings of isolation. Specifically, 30% of school nurses reported feeling less isolated, with 39 of these working with a colleague and 42 working alone. In contrast, of the 70% of school nurses who reported high levels of isolation, 62 worked with a colleague and 125 alone. The p-value for this association was 0.028, which indicates statistical significance at the 95% confidence level.

## Discussion

This aim of this study was to analyze the work situation of school nurses, focusing on the Guidelines, cooperation with the school, the physical work environment, and job satisfaction. The study indicates that many school nurses were satisfied with their jobs and considered their work valuable. However, less than half of the respondents found the Guidelines clear regarding their expected activities in school health services. More years of experience in school health services correlate with feeling more included by school staff, and having a colleague significantly reduces feelings of isolation.

Fifty-three percent of school nurses found the Guidelines somewhat unclear. By the time of this study in 2023, the Guidelines had been in use for 6 years and should have been well implemented and known. A study focusing on the Norwegian Guidelines conducted in 2017 (Waldum-Grevbo, 2018) revealed significant variations in the degree of implementation, which was explained by the Guidelines being newly introduced. This explanation is no longer valid.

School nurses reported a high degree of task prioritization (41%), and nearly 50% felt unable to fulfill their duties as specified in the Guidelines, which aligns with the findings of other studies (ACTIS, 2023; Lassemo & Melby, 2020; Nygård et al., 2024). One reason for this deprioritization could be the way the Guidelines are formulated, as they outline numerous possible tasks with varying levels of recommendation strength (Norwegian Directorate of Health, 2017b). As pointed out by a recent Norwegian study (Nygård et al., 2024), there is a need for the Guidelines to be made clearer, rather than a list of many possible tasks.

This study revealed that 25% of school nurses participated in parent meetings and 41% in educational sessions. Participation in parent meetings and educational sessions could serve as a platform for preventive and health promotion work. These activities are part of the expected tasks in school health services, yet the Guidelines indicate that involvement is only required if the school requests it (Norwegian Directorate of Health, 2017b). Consequently, school nurses become providers, while it is the school staff who request and approve these activities (Waldum-Grevbo, 2018). Preventive efforts and health promotion are fundamental principles for school nurses and are integrated into all activities within school health services (Norwegian Directorate of Health, 2017b; Norwegian Ministry of Health and Care Services, 2018). However, many studies indicate that health-promoting and preventive work is deprioritized due to a focus on other tasks (ACTIS, 2023; Dahl, 2018; Dahl & Clancy, 2015; Jönsson et al., 2019; Lassemo & Melby, 2020; Nygård et al., 2024). The reason that school nurses are unable to carry out all tasks could be found in inconsistent regulations between schools and school health services, resource shortage, unclear Guidelines, or other factors that future research should focus on to provide more clarity.

This study identified themes that school nurses contribute to when they perform teaching lessons. A total of 86.2% reported that they taught pupils about mental health, which emerged as the theme that most school nurses contributed to. In the 2020/21 school year, “Public Health and Life Skills” was introduced as a cross-curricular theme in all obligatory Norwegian schools, with the intention to equip pupils with skills that promote good mental health (Norwegian Directorate for Education and Training, 2019). School nurses have broad competence in public and mental health and the subject fits well within their role in promoting health. The literature indicates that, given the broad scope of this new subject, there is a need for role clarification regarding responsibility for this subject (Pedersen & Wollscheid, 2023), as well as the necessary competencies to teach it (Lystad & Bjørkly, 2018). Currently, this subject is only regulated in the school's curriculum (Norwegian Directorate for Education and Training, 2019), and not specifically in the Guidelines. It is unknown to what extent school staff members and school nurses collaborate to teach this subject.

Effective collaboration between school staff and school nurses is important in all aspects of their cooperative work, not only in teaching. This study indicates that feeling a sense of inclusion requires several years of experience. Key factors for successful cooperation include establishing clear task distribution and understanding each other's roles and responsibilities (Glavin & Kvarme, 2024). Understandably, this comprehension of roles and sense of inclusion improve with longer periods of working together, as time allows individuals to become more familiar with one another. Nonetheless, it is concerning that 52 school nurses with limited experience reported feeling excluded.

The Guidelines state that the school health services should collaborate with schools, emphasizing that school nurses should have meetings with the school's management and educational staff. However, no such requirement exists for school employees to meet with the school nurses. Just over half of the respondents in this study indicated that they had regular meetings with school management to a large extent. When including those who had meetings to some extent, the proportion rises to 82%. These results are similar to those of a 2018 survey that found that 68.5% of school nurses had regular meetings with school management, and a 2023 survey that found that 72% had collaborative meetings with management (Nitter et al., 2023; Waldum-Grevbo, 2018), both of which indicate that there are school nurses who do not have meetings with school management. Without these meetings, it is difficult for school nurses and school staff to be aware of each other's plans.

Actively involving school nurses in school development days could help to synchronize plans, ensuring that school health services align with educational objectives (Glavin & Kvarme, 2024). However, this study found that only16% of participants were extensively involved in development days, despite the Guidelines strongly recommending this involvement (Norwegian Directorate of Health, 2017b). School health services and the schools are working towards the same objective by promoting good mental health and a positive psychosocial environment for all pupils (Norwegian Directorate of Health, 2017b; Norwegian Ministry of Education and Research, 2023). To help achieve this goal, it is essential to establish a strong structure, implement effective routines, and ensure clear role distribution and clarification of each other roles (Glavin & Kvarme, 2024).

Another area where it is important to be aware of each other's plans is in relation to pupils and school nurses’ attendance. This is because school nurses often work in multiple schools, at more than one level, and have other responsibilities within public health in the municipality (Dahl & Blindheim, 2020). School nurses are usually physically working at “their” school only at certain times during the week, and it is not an efficient use of time to arrive at school only to discover that the school is having an off-site day, or that a particular pupil who had a scheduled appointment is absent. School nurses reported that pupils and school staff usually knew when they are present at school; however, the school nurses were seldom notified when the pupils are not at school. This lack of notification could be due to school staff not knowing which pupils the school nurses have appointments with due to the need for confidentiality. In this study, 32% of the school nurses reported that they believed that confidentiality requirements complicated school cooperation. Other research suggests that the problem could instead lie with school nurses themselves, because they prefer to work independently (Federici et al., 2021; Mæland et al., 2021). This aspect, which involves understanding and cooperation around plans, illustrates the difficulty of achieving optimal collaboration. Factors such as confidentiality, the preference to work independently, and disparate regulatory frameworks can also present a challenge and make the collaboration demanding.

In order to perform the activities outlined in the Guidelines, the school nurses require an office that is suitable for school health service activities. The majority of school nurses reported having their own office at school (96%) with only a small number needing to ask to borrow an office. On the other hand, more than 30% of school nurses reported not having a handwashing facility in their school health service office. In many situations, hand sanitizer is just as effective as washing with soap and water (Norwegian Directorate of Health, 2017a); nevertheless, school health services are a health service and handwashing facilities should be easily visible and accessible (Norwegian Directorate of Health, 2017a). There are situations where the school nurse needs to practice first aid and requires access to running water and handwashing facilities. It is interesting to note that previous legislation held schools responsible for providing an office for this purpose (Norwegian Ministry of Health and Care Services, 1984); however, this is not regulated in any legislation today (Norwegian Directorate of Health, 2017b; Norwegian Ministry of Health and Care Services, 2018).

The location of the school health service office within the school building is also important. The Guidelines state that the school health service should have a central location and be accessible to students for drop-in appointments so that they can get an appointment when they need it (Norwegian Directorate of Health, 2017b). In this study, 14% of the school nurses reported not having time for drop-in appointments, and if those who only had a limited amount of drop-in time are included, the proportion rises to almost 50%. Additionally, 12% of school nurses in this study reported not having an office in a central location within the school building, which is important both for collaboration (Waldum-Grevbo, 2018) and the accessibility of the school health service for pupils (Norwegian Directorate of Health, 2017b).

Beyond the implementation of the Guidelines, school nurses in Norway have continually been in a position where new proposals for the content and purpose were introduced by central authorities. These have ranged from suggestions to merge EPS with school health services (Meld. St. 6 (2019 −2020)), to grant school nurses the right to refer children and adolescents to outpatient psychiatric services at the hospital (Meld.St.23 (2022–2023)), and to expand the school health service's responsibilities for diagnosis, treatment, and follow-ups (Norwegian Ministry of Health and Care Services, 2017, 2020). In addition to being subjected to constantly changing proposals, many school nurses were reassigned to other tasks within municipalities during the COVID- 19 pandemic, despite the undiminished need for assistance of children and adolescents (Melby et al., 2020). These sorts of problems could contribute to the reported perception among school nurses that school staff do not fully understand the nurse's role in school health services. To understand each other's roles, clear job descriptions are necessary; however, many school nurses reported a lack of clarity in the Guidelines.

Although development is important, it can be difficult to develop in a work situation where tasks are easily reassigned when the municipality needs health personnel for other tasks, and when the job description is frequently under review for reassessment. These factors can create uncertainty for both the school nurses themselves and the school staff.

## Limitations and Future Research

This study provides broad insights into the situation of school nurses in school health services. However, there are also some limitations. There is some weakness in external validity in terms of generalizability. In 2023, Norway had 356 municipalities; here, a total of 120 municipalities were invited to participate in the survey, and the school nurses who actually participated were from 72 municipalities. In other words, many municipalities were not represented in this study. There is also some weakness in internal validity because a non-validated questionnaire was used. However, to ensure the reliability of the questionnaire, it was tested by 10 school nurses before it was used for this study.

A qualitative follow-up study is needed for a more in-depth investigation of the underlying factors for the results in this study. This future study should also incorporate the perspectives of both school staff and pupils to ensure a comprehensive understanding. Additionally, research could be done to examine the influence of regulations on the collaboration between schools and school health services, from the perspectives of both those actors.

## Conclusions

This study provides valuable insights into the work situation of school nurses within Norwegian school health services. Certain areas require further research, as there are indications of needs for improvement. The majority of school nurses who reported feelings of isolation were those who worked alone. Although this finding is not unexpected, the staffing authorities should take this factor into account and strive to ensure that school nurses have colleagues and can work collaboratively within a team when they make plans for the school health services.

The integration of school nurses into the school staff is clearly a process that takes time, as those with most experience also felt the most included. Although this relationship seems intuitive, further exploration is needed to understand why some school nurses do not feel included.

Although schools and school health services should ideally cooperate closely, imbalances can emerge when legislation primarily governs only one service. The Guidelines for school health services is the only regulation that includes provisions for this cooperation. This could lead to situations where school staff are not fully aware of the Guidelines, tasks, and role definitions pertaining to the school health services. The lack of clarity could potentially hinder effective cooperation between the two, as the individual roles and responsibilities might not be clearly understood or evenly distributed.

The results in this study reveal that a significant number of school nurses found the Guidelines that regulate their work to be unclear. This lack of clarity can lead to uncertainty and varied practices, which contradicts the intended purpose of the Guidelines. The Guidelines need to be clearer and more precise if the aim is to standardize school health services and ensure a common understanding of roles and responsibilities among all school nurses.

Despite these challenges, this study confirms that, in general, school nurses perceive their work as valuable and that they possess the necessary knowledge to perform their tasks. It is important for pupils, to have school nurses who are satisfied in their work and who feel that they are adequately equipped with the requisite knowledge.
